# Supplementary material for: SLR1 inhibits MOC1 degradation to coordinate tiller number and plant height in rice
Source: Nat Commun. 2019 Jun 21;10:2738. doi: 10.1038/s41467-019-10667-2 (PMC6588547; doi:10.1038/s41467-019-10667-2)
Supplement: Supplementary file 1 — Supplementary Information [file 41467_2019_10667_MOESM1_ESM.pdf]

Supplementary Information

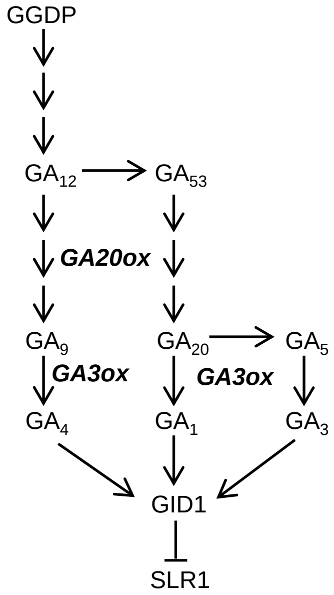

Supplementary Figure 1. Schematic diagram of GA biosynthesis and signaling pathways.

GA3ox, GA 3-oxidase; *GA20ox, GA20-oxidase*; GGDP, geranylgeranyl diphosphate; GID1, GIBBERELLIN-INSENSITIVE DWARF 1; SLR1, SLENDER RICE 1.

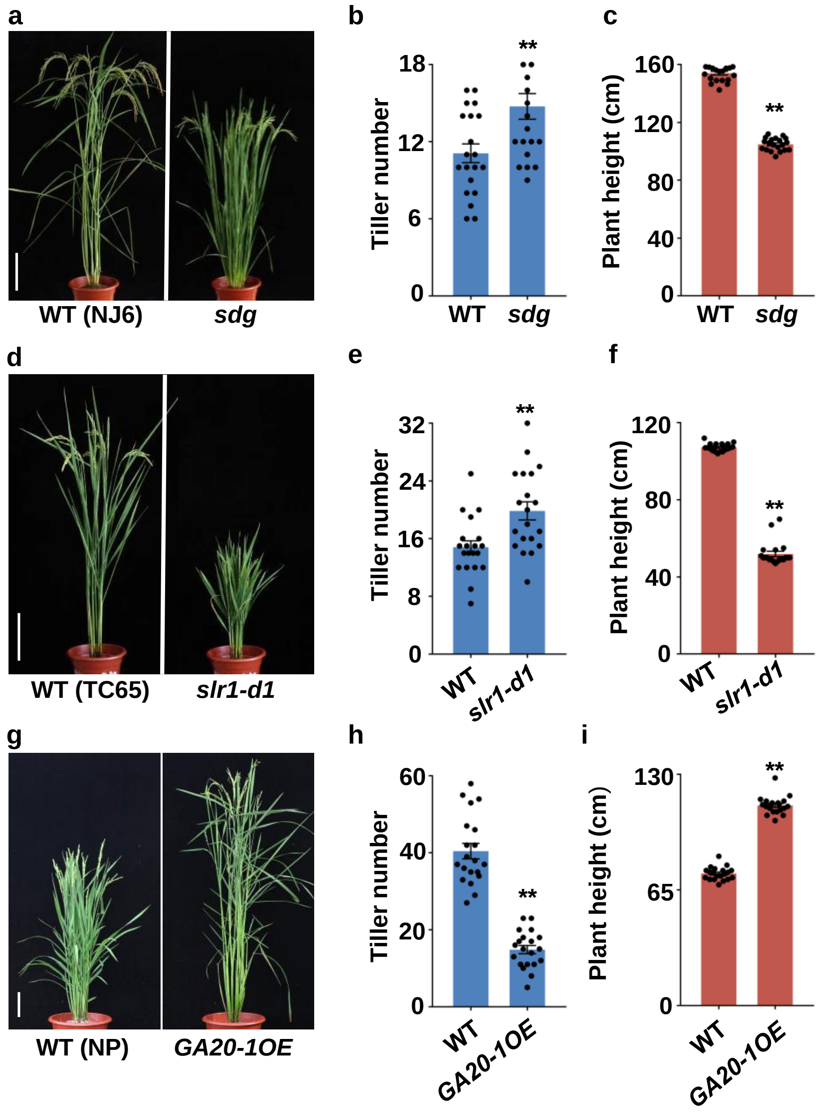

Supplementary Figure 2. Tiller number is negatively correlated with plant height in GA-related mutants and transgenic plants.

(a) Appearance of the WT and *sdg* mutant at the heading stage. Scale bar, 20 cm. (b) and (c) Quantification of the tiller number and plant height shown in (a). Asterisks indicate significant difference to WT. (d) Appearance of the WT and *slr1-d1* mutant at the heading stage. Scale bar, 20 cm. (e) and (f) Quantification of the tiller number and plant height shown in (d). (g) Appearance of the WT and *GA20ox-1* overexpressing (*GA20-1OE)* transgenic plants at the heading stage. Scale bar, 20 cm. (h) Tiller number and (i) height of the plants shown in (g). Asterisks indicate significant difference to the WT (two-tailed Student's *t*-test, $^{**}p < 0.01$; mean ± s.e.m., n = 20 in (**b**), (**c**) , (**e**), (**f**), (**h**), and (**i**)). Source data are provided as a Source Data file.

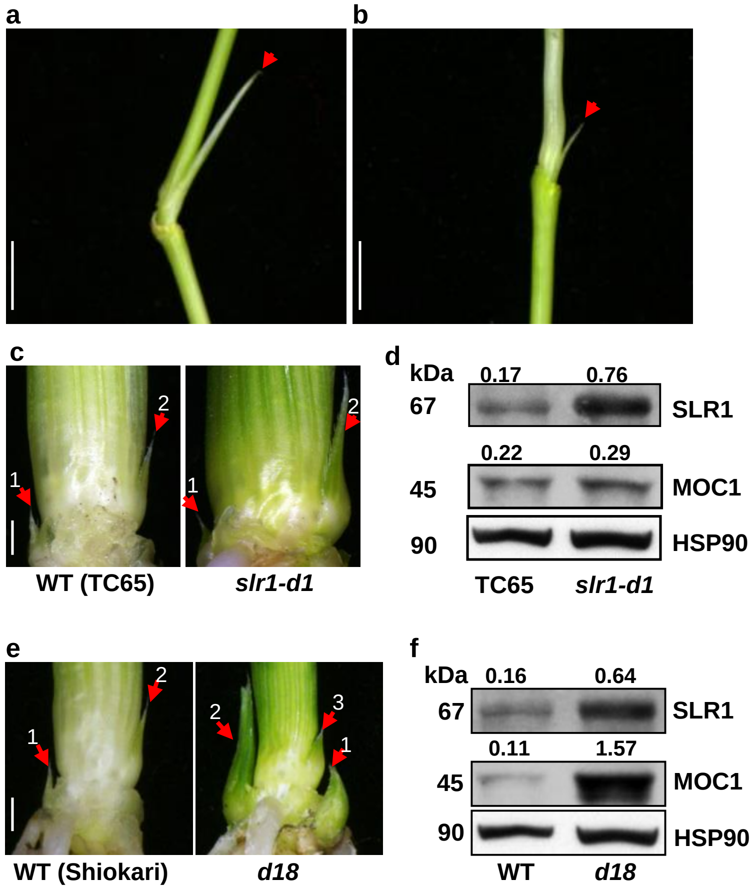

Supplementary Figure 3. Axillary buds and MOC1 protein in GA-related mutants. (a) Third and (b) fourth axillary buds of *slr1*. Arrows indicate axillary buds. Scale bar, 1 mm. (c) Dissections of the WT (TC65) and *slr1-d1* seedlings at 30 days after germination (DAG). Arrows indicate axillary buds. Scale bar, 1 mm. (d) Protein levels of SLR1 and MOC1 in extracts of shoot bases (0 to 0.5 cm) of the WT and *slr1-d1* seedlings, determined by protein immunoblotting. HSP90, loading control. Values above panels indicate signal strength for SLR1 and MOC1 in arbitrary units determined by densitometry. (e) View of shoot base of the WT (Shiokari) and *d18* seedlings at 30 DAG. Arrows indicate axillary buds. Scale bar, 1 mm. (f) Protein levels of SLR1 and MOC1 in extracts of the WT and *d18* seedlings, determined by protein immunoblotting. HSP90, loading control. Values above panels indicate signal strength for SLR1 and MOC1 in arbitrary units determined by densitometry. The full scans of immunoblots are shown in Supplementary Fig. 12.

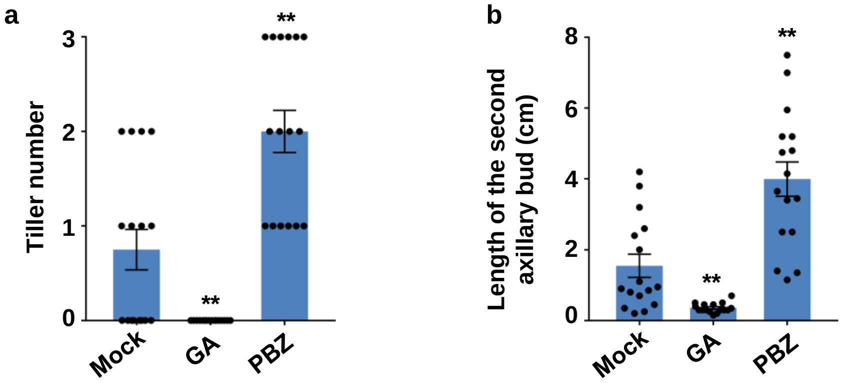

Supplementary Figure 4. Effect of GA and PBZ treatments on tiller bud outgrowth. (a) Quantification of the tiller number shown in Fig 2 (e). Asterisks indicate significant difference to the WT. (b) Length of the second axillary buds shown in Fig 2 (e). Asterisks indicate significant difference to the WT (two-tailed Student's *t*-test, $^{**}p < 0.01$; mean $\pm$ s.e.m., n = 16). Source data are provided as a Source Data file.

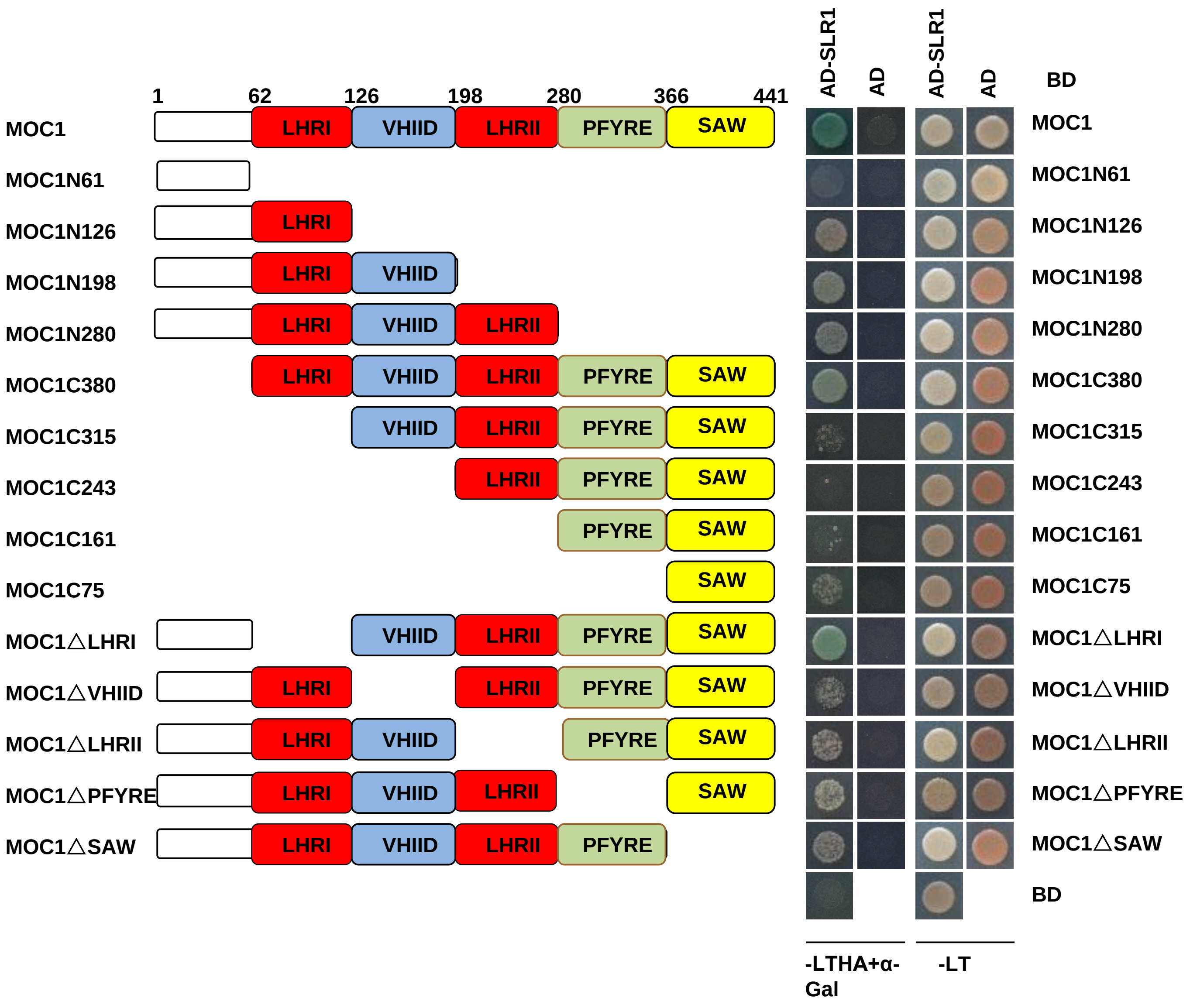

Supplementary Figure 5. ***In vitro*** **Y2H assay showing MOC1 motifs interacting with SLR1.** (left) Schematic representation of MOC1 and its conserved motifs shown by color: Red, LHRI or LHRII; blue, VHIID; green, PFYRE; and yellow, SAW. Numbers at the top indicate the position of amino acids. (right) Y2H analyses of the interactions between SLR1 and MOC1 derived proteins shown in (left). Transformed yeast cells were grown on synthetic complete medium lacking Trp and Leu (-LT), or synthetic complete medium lacking Trp, Leu, His and Ade (-LTHA); α-Gal, 5-Bromo-4-chloro-3-indoxyl-α-D-galactopyranoside. The –LTHA+α-Gal medium contained 40 μg·mL^{-1} X-α-Gal. AD, GAL4 activation domain; BD, GAL4 DNA binding domain. Triangles indicate deletion.

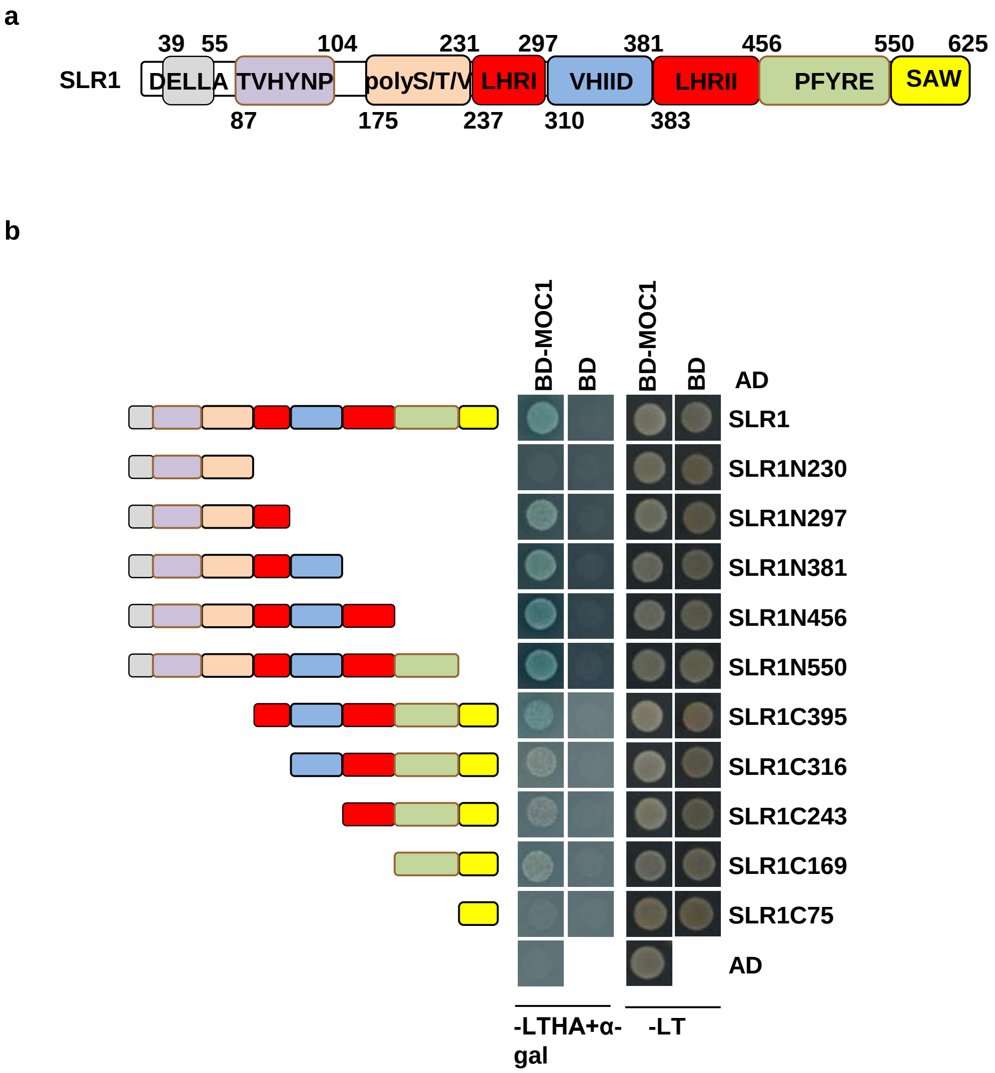

a
39
55
104
231
297
381
456
550
625
SLR1
DELLA
TVHYNP
polyS/T/V
LHRI
VHIID
LHRII
PFYRE
SAW
87
175
237
310
383
b
BD-MOC1
BD
BD-MOC1
BD
AD
SLR1
SLR1N230
SLR1N297
SLR1N381
SLR1N456
SLR1N550
SLR1C395
SLR1C316
SLR1C243
SLR1C169
SLR1C75
AD
-LTHA+α-gal
-LT

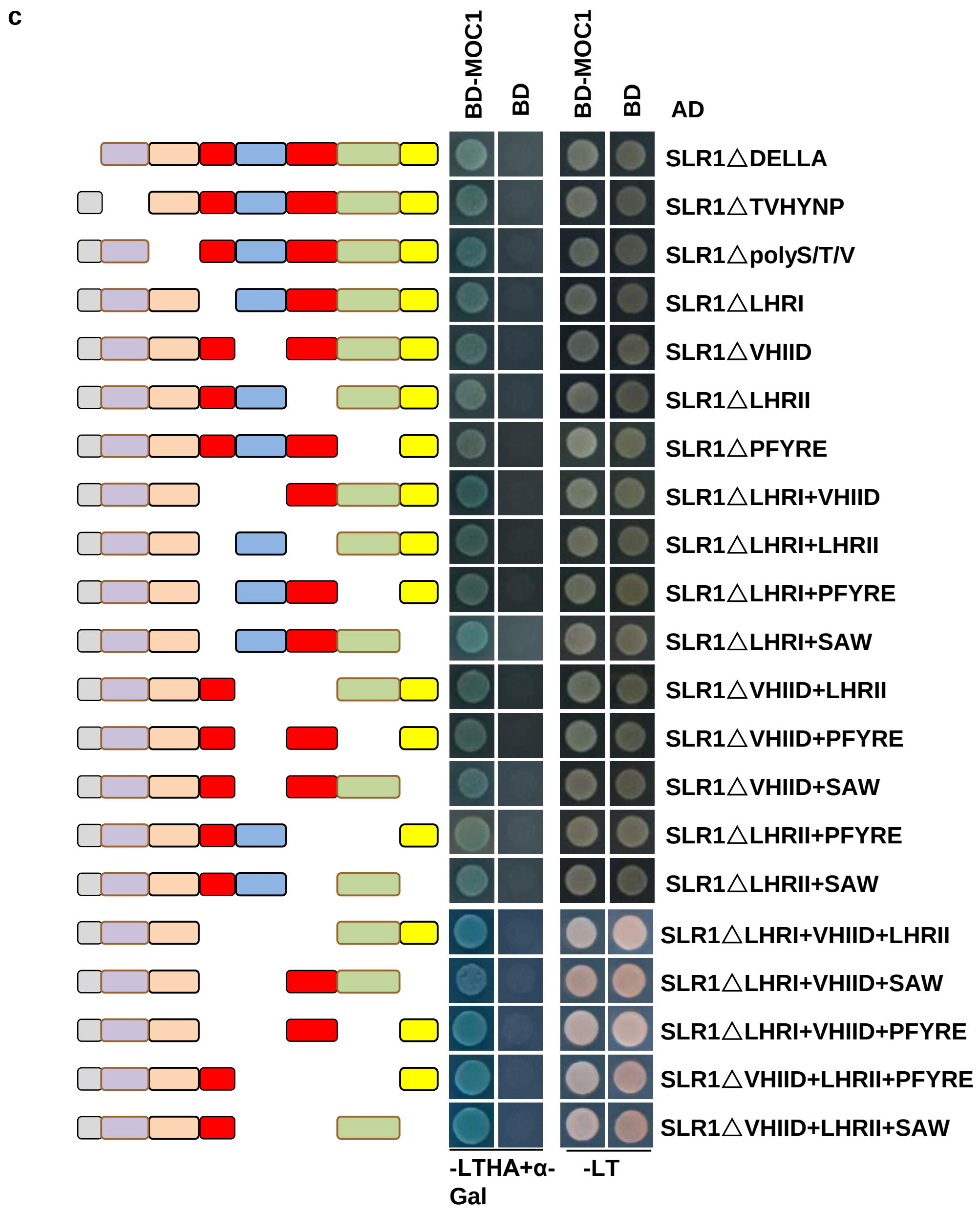

Supplementary Figure 6. ***In vitro*** **Y2H assays showing SLR1 motifs interacting with MOC1.** (a) Schematic representation of SLR1 and its conserved motifs shown by color: Gray, DELLA; purple, TVHYNP; orange, polyS/T/V; red, LHRI or LHRII; blue, VHIID; green, PFYRE and yellow, SAW. Numbers at the top indicate the position of amino acids. (b) Y2H analyses of the interaction between MOC1 and derivatives of SLR1 shown in the left. (c) Y2H analyses of the interaction between MOC1 and derivatives of SLR1 shown in the left. Transformed yeast cells were grown on synthetic complete medium lacking Trp and Leu (-LT), or synthetic complete medium lacking Trp, Leu, His and Ade (-LTHA); α-Gal, 5-Bromo-4-chloro-3-indoxyl-α-D-galactopyranoside, which is a chromogenic substrate for α-galactosidase. The –LTHA+α-Gal medium contained 40 $\mu g \cdot mL^{-1}$ X-α-Gal. AD, GAL4 activation domain; BD, GAL4 DNA binding domain. Triangles indicate deletion.

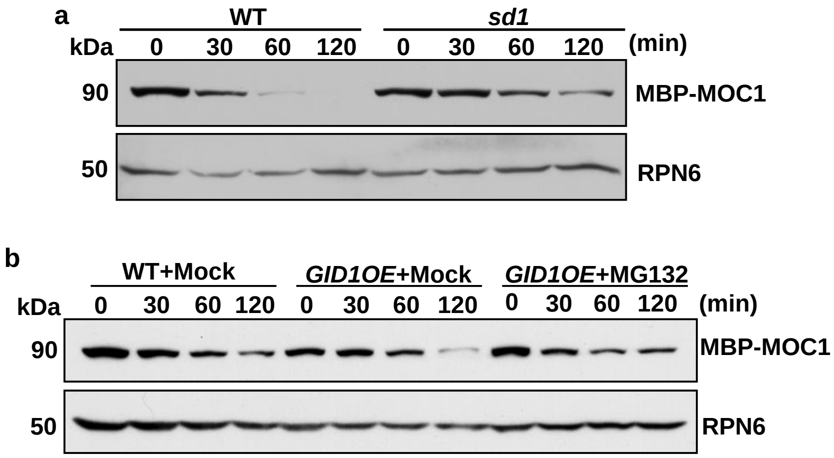

Supplementary Figure 7. The degradation of MOC1 is affected by GA biosynthesis signaling and MG132.

(a) *In vitro* cell-free protein degradation assay, showing degradation of MBP-MOC1 in extracts from the WT and *sd1*. Immunoblots were probed with anti-MBP (α-MBP). Ribosomal protein 6 (RPN6), loading control. (b) *In vitro* cell-free protein degradation assay, showing MG132 could partially inhibit the degradation of MBP-MOC1 in extracts from *GID1OE* transgenic plants. Immunoblots were probed as in (a). Source data are provided as a Source Data file.

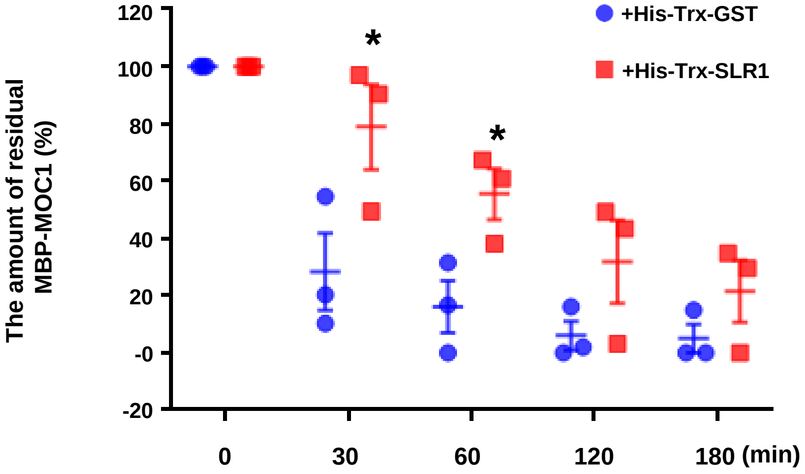

Supplementary Figure 8. His-Trx-SLR1 inhibits the degradation of MOC1 in cell-free protein degradation assay.

Relative amounts of proteins in Fig. 4c and other two biological replicates were determined by densitometry normalized to RPN6. Asterisks indicate significant difference between +His-Trx-GST and +His-Trx-SLR1 at each time point (two-tailed Student's *t*-test, $*p < 0.05$; mean ± s.e.m., n = 3). Source data are provided as a Source Data file.

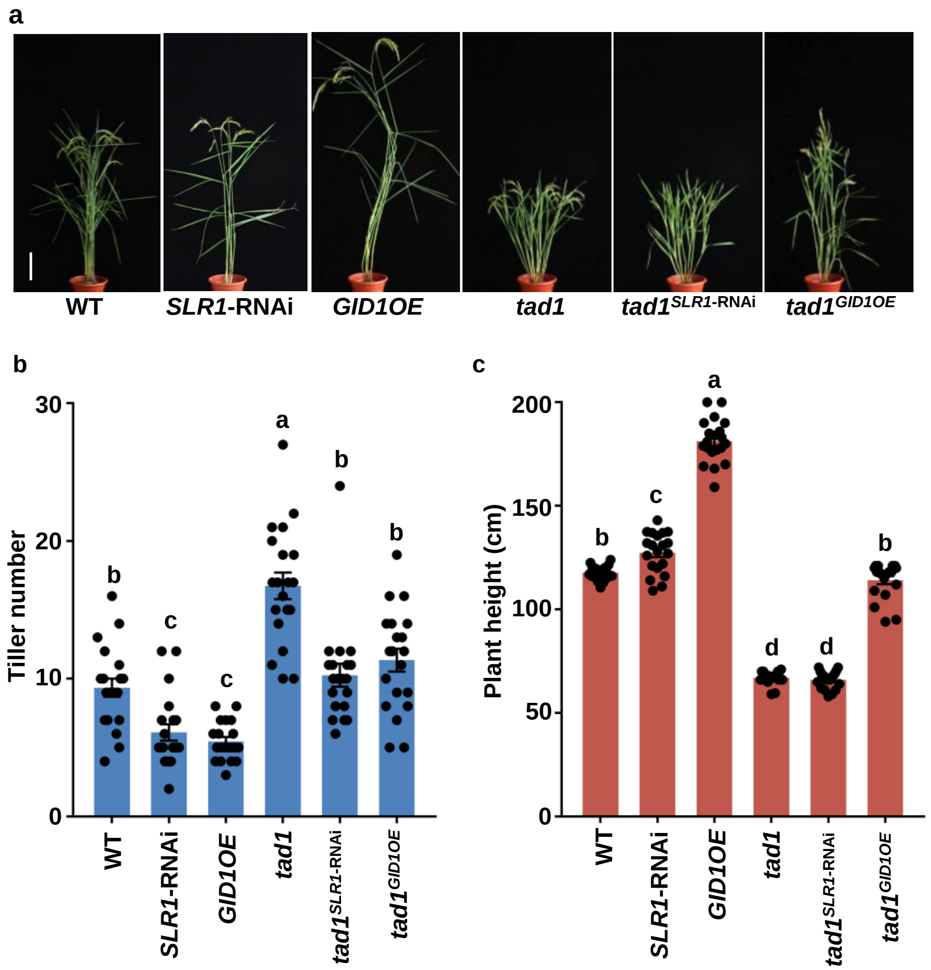

Supplementary Figure 9. Knock-down *SLR1* and overexpressing-*GID1* can rescue the tiller number of *tad1*.

(a) Phenotypes of *SLR1*-RNAi, *GID1OE* in the WT (LS), and *tad1* at the heading stage. Scale bar, 20 cm. (b) Tiller number and (c) height of plants shown in (a). Different lowercase letters indicate significant differences (Tukey's HSD test. $p < 0.05$; mean $\pm$ s.e.m., n = 20). Source data are provided as a Source Data file.

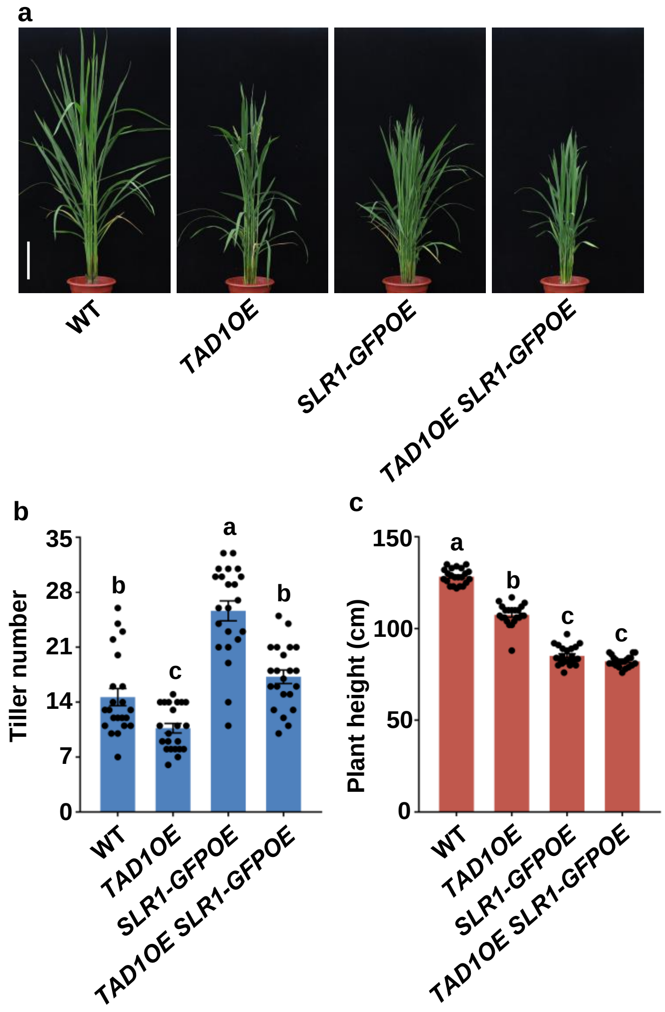

Supplementary Figure 10. Analysis phenotypes of *TAD1OE SLR1-GFPOE*.
(a) Phenotypes of WT (LS), *TAD1OE*, *SLR1-GFPOE* and *TAD1OE SLR1-GFPOE* plants. Scale bar, 20 cm. (b) Tiller number and (c) height of plants shown in (a). Different lowercase letters indicate significant differences (Tukey's HSD test. $p < 0.05$; mean ± s.e.m., n = 22). Source data are provided as a Source Data file.

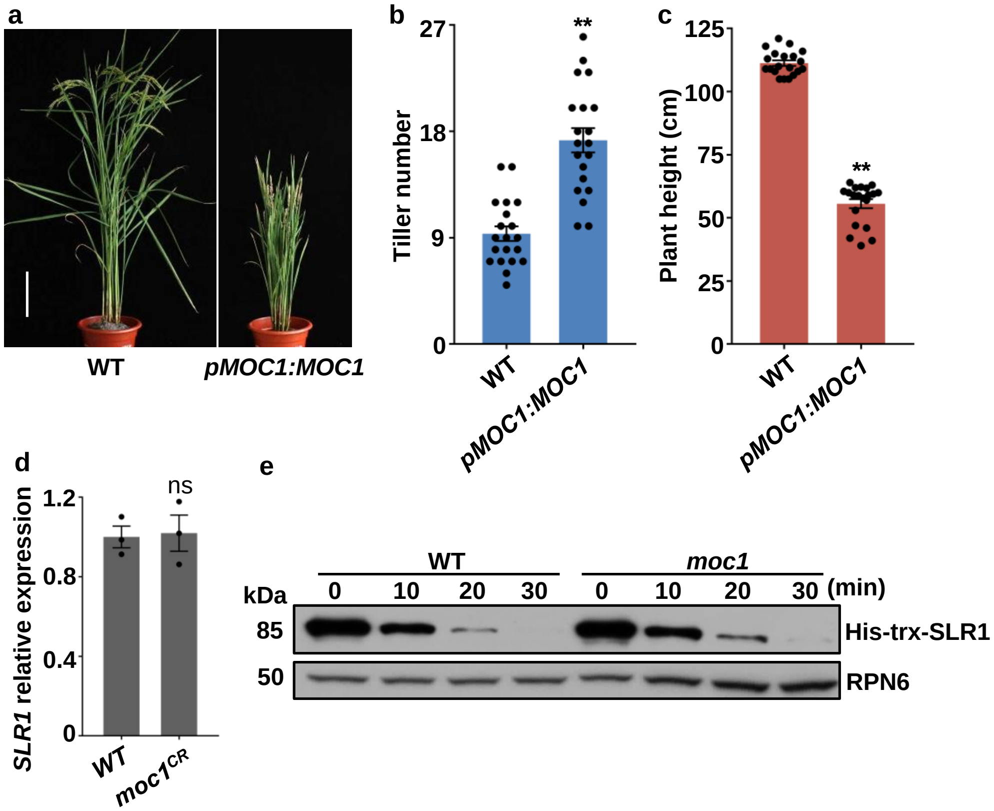

Supplementary Figure 11. MOC1 regulates plant height not through mediating SLR1 transcription or degradation.

(a) Phenotypes of WT (LS) and p*MOC1:MOC1* plants at the heading stage. Scale bar, 20 cm. (b) Tiller number and (c) height of plants shown in (a). Asterisks indicate significant difference to the WT (two-tailed Student's *t*-test, $^{**}p < 0.01$; mean $\pm$ s.e.m., n = 20). (d) The expression level of *SLR1* in shoot bases (0 to 0.5 cm) in one-month-old seedlings of WT and $moc1^{CR}$. The ns indicates no significant difference to WT (two-tailed Student's *t*-test; mean $\pm$ s.e.m., n = 3).(e) Degradation of His-trx-SLR1 in WT and *moc1*. RPN6, loading control. Source data are provided as a Source Data file.

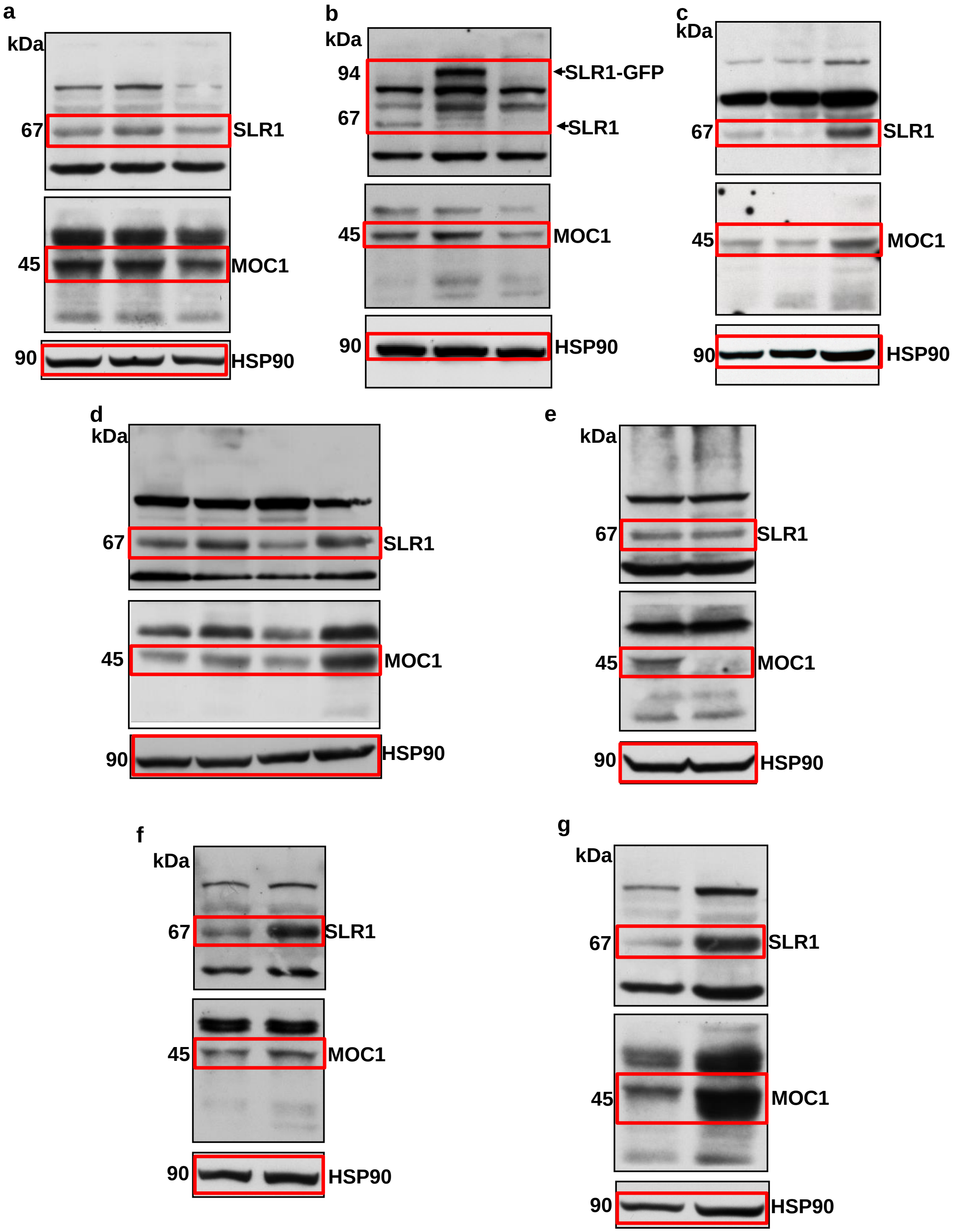

Supplementary Figure 12. Full scans of immunoblots in Figures 2b (a), 2d (b), 2f (c), 4g (d), 5a (e) and Supplementary Figure 3b (f), 3f (g).
Molecular weight markers are indicated in kDa.

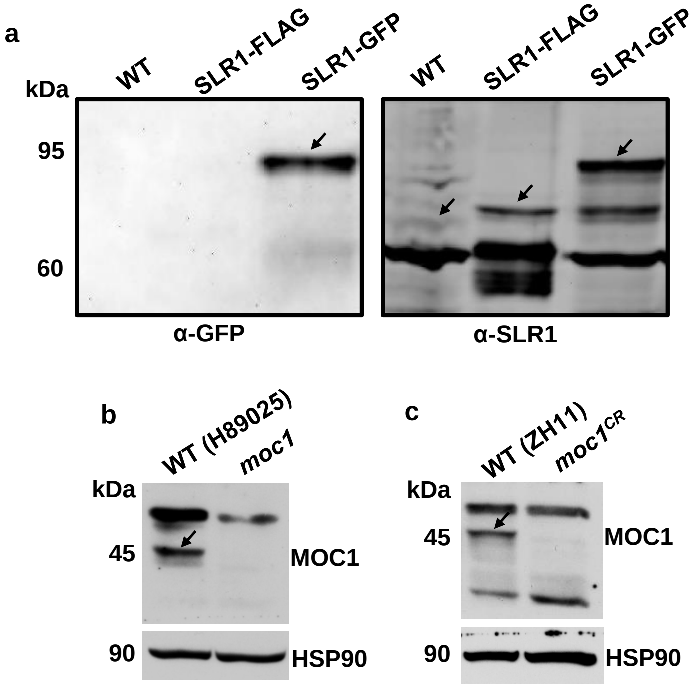

Supplementary Figure 13. Determination of SLR1 and MOC1 antibody specificity.
(a) Determination of SLR1 antibody specificity by SLR1-FLAG and SLR1-GFP protein extracts from rice protoplasts. Right, anti-SLR1 (1:1,000); left, anti-GFP (1:5,000). (b) and (c) Determination of MOC1 antibody specificity using protein extracts from *moc1* and *moc1*^{CR} mutants.

Supplementary Table 1. Information of the mutants and the transgenic plants used in this study.

Name	Wild-type	Information
d18	Shi.	*dwarf18* with defective *GA3ox-2*
GA20-1OE	Nip.	*GA20ox-1* overexpression plant
sdg	NJ6	*gid1* with point mutation cDNA G (493) to A in *GID1*
slr1-d1	TC65	*slender* with cDNA T (317) to A in *SLR1*
moc1	H89025	*moc1* with 1.9-kb retrotransposon insertion at 948 of *MOC1*
$gid1^{CR}$	ZH11	*gid1* with 1-bp insertion at 103 of *GID1*
$moc1^{CR}$	ZH11	*moc1* with 1-bp deletion at 654 of *MOC1*
sd1	ZH11	*semi-dwarf1* with 7-bp deletion at 546-552 of *GA20ox-2*
slr1	ZH11	*slender* with cDNA T (737) to C in *SLR1*
GID1OE	LS	*GID1* overexpression plant
pMOC1:MOC1	LS	*MOC1* overexpression plant
SLR1-GFPOE	LS	*SLR1* overexpression plant
SLR1-RNAi	LS	*SLR1* knock-down plant
tad1	LS	*tad1* with point mutation cDNA G (717) to A in *TAD1*
TAD1OE	LS	*TAD1* overexpression plant

Abbreviations: Shi., Shiokari; Nip., Nipponbare; NJ6, Nanjing6; TC65, Taichang65; ZH11, Zhonghua11; LS, Lansheng.

Supplementary Table 2. Primers used in this study.

Construct	Primer	Sequence (5'-3')
SLR1-GFPOE	SLR1-GFPOESmaF	TCCCCCGGGCATGAAGCGCGAGTACCAA
	SLR1-GFPOEXbaR	GCTCTAGACGCCGCGGCGACGCGCCATG
SLR1-RNAi	SLR1-RNAiBamF	GCGCGGATCCCGCAGCCGGACGAGACCGACGCCTTGC
	SLR1-RNAiKpnR	GCGGTACCAGCTCGGCCTGGCCGGAGCT
	SLR1-RNAiSacF	GCGCGAGCTCCGCAGCCGGACGAGACCGACGCCTTGC
	SLR1-RNAiSpeR	GCACTAGTAGCTCGGCCTGGCCGGAGCT
GID1OE	GID1OE-BamHF	CGGGATCCATGGCCGGCAGCGACGAGGTCAA
	GID1OE-SpeR	GACTAGTCTAGTAGTAGAGGTTAGCGTTGA
Anti-SLR1	Anti-SLR1HindF	CCCAAGCTTATGAAGCGCGAGTACCAAGAAG
	Anti-SLR1EcoRR	GGAATTCTCACATGGCGCCGCCCTGGGACGCGGCCAG
CC-SLR1/*35S-SLR1*	35S-SLR1XbaF	GCTCTAGAATGAAGCGCGAGTACCAAGA
	35S-SLR1KpnR	GGGGTACCCGCCGCGGCGACGCGCCATG
AD-SLR1	AD-SLR1NdeF	GGAATTCCATATGATGAAGCGCGAGTACCAAGAAG
	AD-SLR1EcoRR	GGAATTCCGCCGCGGCGACGCGCCA
BD-MOC1	BD-MOC1F	CGGGATCCATGCTCCGGTCACTCCACTC
	BD-MOC1R	CGGAATTCCTACGACGACGACGGCTGCCAC
MBP-MOC1	MBP-MOCEcoRF	CCCGGAATTCATGCTCCGGTCACTCCACTC
	MBP-MOCHindR	CCCGGTACCCTACGACGACGACGGCTGCC
BD-MOC1N61	MOC1N61R	CGGGATCCCAGCAGGTCCGCGCACGC
BD-MOC1N126	MOC1N126R	CGGGATCCCGCCCCGGACGACGCCGG
BD-MOC1N198	MOC1N198R	GGGGTACCGGCGCCGGCGCCGGTGACGCGGACCT
BD-MOC1N280	MOC1N280R	CGGGATCCGTGGCCGGCCAGGTTGTG
BD-MOC1N366	MOC1N366R	CGGGATCCGGAGGGCCCCACCGCGGC
BD-MOC1C380	MOC1C380F	GGAATTCCAGAGGGGGGACCTGCCG
BD-MOC1C315	MOC1C315F	GGAATTCCAGAGGGGGGACCTGCCG
BD-MOC1C243	MOC1C243F	GGAATTCGACCGCGACACCCTCCTC
BD-MOC1C161	MOC1C161F	GGAATTCGACGAGCTCGCCGCGTTC
BD-MOC1C75	MOC1C75F	GGAATTCGGCGGCCGGTGGTGGCGC
BD⊿MOC1⊿LHRI	MOC1△LHR1F	GCGTGCGCGGACCTGCTGTACCTGGCGTTCAACCAG
	MOC1△LHR1R	CTGGTTGAACGCCAGGTACAGCAGGTCCGCGCACGC
BDMOC1⊿VHIID	MOC1△VHIIDF	CCGGCGTCGTCCGGGGCGGACCGCGACACCCTCCTC
	MOC1△VHIIDR	GAGGAGGGTGTCGCGGTCCGCCCCGGACGACGCCGG
BDMOC1⊿PFYRE	MOC1△PFYREF	CACAACCTGGCCGGCCACGGCGGCCGGTGGTGGCGC
	MOC1△PFYRER	GCGCCACCACCGGCCGCCGTGGCCGGCCAGGTTGTG
BDMOC1⊿LHRII	MOC1△LHRIIF	GTCACCGGCGCCGGCGCCGACGAGCTCGCCGCGTTC
	MOC1△LHRIIR	GAACGCGGCGAGCTCGTCGGCGCCGGCGCCGGTGAC
AD-SLR1N230	SLR1N230R	GGAATTCCACAACCGGCACGGCGGG
AD-SLR1N297	SLR1N297R	GGAATTCGCGGAAGCGGTACACGCG
AD-SLR1N381	SLR1N381R	GGAATTCGGGGCCGACGCCGGTGAG
AD-SLR1N456	SLR1N456R	GGAATTCGGGCTGCGCGAGCAGCCG
AD-SLR1N550	SLR1N550R	GGAATTCCTCCGCGCCCTCGCACGC

Supplementary Table 2. Primers used in this study. (Continued from the previous page)

Construct	Primer	Sequence (5'-3')
AD-SLR1C395	SLR1C395F	GGAATTCCATATGGTGGTTGACACGCAGGAG
AD-SLR1C316	SLR1C316F	GGAATTCCATATGGACCTTCTGCACGCCCAC
AD-SLR1C243	SLR1C243F	GGAATTCCATATGCAGCCGGACGAGACCGAC
AD-SLR1C169	SLR1C169F	GGAATTCCATATGGGCGCGCTGGAGAAGGTC
AD-SLR1C75	SLR1C75F	GGAATTCCATATGCGCACGGAGCGCCACGAG
AD-SLR1⊿DEA	SLR1∆DEAF	GGGGAGGAGGAGGACGTCGACGTCGCGCAGAAGCTG
	SLR1∆DEAR	CAGCTTCTGCGCGACGTCGACGTCCTCCTCCTCCCC
AD-SLR1⊿TVP	SLR1∆TVPF	GACGGGTTCGTGTCGCACAGCATGCTTTCCGAGCTC
	SLR1∆TVPR	GTTGAGCTCGGAAAGCATGCTGTGCGACACGAACCCGTCATC
AD-SLR1⊿polyS/T/V	SLR1∆polySF	GACCCGTCGGCTGCTGACGGGATCCGGCTGGTGCAC
	SLR1∆polySR	GTGCACCAGCCGGATCCCGTCAGCAGCCGACGGGTC
AD-SLR1⊿LHR1	SLR1∆LHR1F	CCCGCCGTGCCGGTTGTGCCCGCGGACAGCACCCTC
	SLR1∆LHR1R	GAGGGTGCTGTCCGCGGGCACAACCGGCACGGCGGG
AD-SLR1⊿VHIID	*AD-SLR1⊿VHIID*F	CTCCTCGACGCCGCCTTCGCCCCGCAGCCGGACGAGACCGAC
	*AD-SLR1⊿VHIID*R	GTCGGTCTCGTCCGGCTGCGGGGCGAAGGCGGCGTCGAGGAG
AD-SLR1⊿LHRII	*AD-SLR1⊿LHRII*F	ACCGGCGTCGGCCCCCCGGGCGCGCTGGAGAAGGTC
	*AD-SLR1⊿LHRII*R	GACCTTCTCCAGCGCGCCCGGGGGGCCGACGCCGGT
AD-SLR1⊿PFYRE	*AD-SLR1⊿PFYRE*F	CGGCTGCTCGCGCAGCCCCGCACGGAGCGCCACGAG
	*AD-SLR1⊿PFYRE*R	CTCGTGGCGCTCCGTGCGGGGCTGCGCGAGCAGCCG
AD-SLR1⊿SAW	*AD-SLR1⊿SAW*F	GTGGCGTGCGAGGGCGCGGAGGCCGCGGCGGAATTCC
	*AD-SLR1⊿SAW*R	GGAATTCCGCCGCGGCCTCCGCGCCCTCGCACGCCAC
AD-SLR1⊿LHRI+VHIID	SLR1∆L1VF	CCCGCCGTGCCGGTTGTGCCGCAGCCGGACGAGACC
	SLR1∆L1VR	GGTCTCGTCCGGCTGCGGCACAACCGGCACGGCGGG
AD-SLR1⊿VHIID+LHRII	SLR1∆VL2F	CTCGACGCCGCCTTCGCCGGCGCGCTGGAGAAGGTC
	SLR1∆VL2R	GACCTTCTCCAGCGCGCCGGCGAAGGCGGCGTCGAG
ADSLR1⊿LHRII+PFYRE	SLR1∆L2PF	ACCGGCGTCGGCCCCCCGCGCACGGAGCGCCACGAG
	SLR1∆L2PR	CTCGTGGCGCTCCGTGCGCGGGGGGCCGACGCCGGT
GID1-CRISPR	*GID1-CRISPRF*	CAGTGTCGTACAACATTCTGCGG
	GID1-CRISPRR	AACCCGCAGAATGTTGTACGACA
MOC1-CRISPR	*MOC1-CRISPRF*	CAGGAAGTGGAAGGGGAGGTGGA
	MOC1-CRISPRR	AACTCCACCTCCCCTTCCACTTC
	QSLR1F	GCTCCAATGCCTACAAACA
	QSLR1R	TTCTCCTCCACCCGGTAG
	QOsUbqF	AACCAGCTGAGGCCCAAGA
	QOsUbqR	ACGATTGATTTAACCAGTCCATGA
